# Exploring the influence of atmospheric CO_2_ and O_2_ levels on the utility of nitrogen isotopes as proxy for biological N_2_ fixation

**DOI:** 10.1128/aem.00574-24

**Published:** 2024-09-25

**Authors:** Nicola Wannicke, Eva E. Stüeken, Thorsten Bauersachs, Michelle M. Gehringer

**Affiliations:** 1 Leibniz Institute for Plasma Science and Technology e.V., Greifswald, Germany; 2 School of Earth & Environmental Sciences, University of St. Andrews, St. Andrews, United Kingdom; 3 Institute of Organic Biochemistry in Geo-Systems, RWTH Aachen University, Aachen, Germany; 4 Department of Microbiology, University of Kaiserslautern-Landau (RPTU), Kaiserslautern, Germany; University of Wisconsin-Madison, Madison, Wisconsin, USA

**Keywords:** nitrogen isotopic fractionation, biological nitrogen fixation, cyanobacteria, carbon:nitrogen ratios, nodularin

## Abstract

Biological N_2_ fixation (BNF) is traced to the Archean. The nitrogen isotopic fractionation composition (δ^15^N) of sedimentary rocks is commonly used to reconstruct the presence of ancient diazotrophic ecosystems. While δ^15^N has been validated mostly using organisms grown under present-day conditions; it has not under the pre-Cambrian conditions, when atmospheric *p*O_2_ was lower and *p*CO_2_ was higher. Here, we explore δ^15^N signatures under three atmospheres with (i) elevated CO_2_ and no O_2_ (Archean), (ii) present-day CO_2_, and O_2_ and (iii) future elevated CO_2_, in marine and freshwater, heterocytous cyanobacteria. Additionally, we augment our data set from literature for more generalized dependencies of δ^15^N and the associated fractionation factor epsilon (*ε* = δ^15^N_biomass_ – δ^15^N_N2_) during BNF in Archaea and Bacteria, including cyanobacteria, and habitats. The *ε* ranges between 3.70‰ and −4.96‰ with a mean *ε* value of −1.38 ± 0.95‰, for all bacteria, including cyanobacteria, across all tested conditions. The expanded data set revealed correlations of isotopic fractionation of BNF with CO_2_ concentrations, toxin production, and light, although within 1‰. Moreover, correlation showed significant dependency of *ε* to species type, C/N ratios and toxin production in cyanobacteria, albeit it within a small range (−1.44 ± 0.89‰). We therefore conclude that δ^15^N is likely robust when applied to the pre-Cambrian-like atmosphere, stressing the strong cyanobacterial bias. Interestingly, the increased fractionation (lower *ε*) observed in the toxin-producing *Nodularia* and *Nostoc* spp. suggests a heretofore unknown role of toxins in modulating nitrogen isotopic signals that warrants further investigation.

**IMPORTANCE:**

Nitrogen is an essential element of life on Earth; however, despite its abundance, it is not biologically accessible. Biological nitrogen fixation is an essential process whereby microbes fix N_2_ into biologically usable NH_3_. During this process, the enzyme nitrogenase preferentially uses light ^14^N, resulting in ^15^N depleted biomass. This signature can be traced back in time in sediments on Earth, and possibly other planets. In this paper, we explore the influence of *p*O_2_ and *p*CO_2_ on this fractionation signal. We find the signal is stable, especially for the primary producers, cyanobacteria, with correlations to CO*_2_*, light, and toxin-producing status, within a small range. Unexpectedly, we identified higher fractionation signals in toxin-producing *Nodularia* and *Nostoc* species that offer insight into why some organisms produce these N-rich toxic secondary metabolites.

## INTRODUCTION

Biological N_2_ fixation (BNF) is a fundamental metabolism that controls the size and diversity of Earth’s biosphere. Only a subset of Bacteria and Archaea, so-called diazotrophs, possess the needed enzymatic machinery to break the triple bond of the N_2_ molecule and convert it into bioavailable ammonia (1). While several abiotic sources of bioavailable nitrogen have been identified, including lightning discharge (2–4), volcanism (5), photolysis (6), and hydrothermal reduction reactions (7–9), the size of these fluxes is poorly constrained and would likely have been heterogeneous over space and time. The origin of diazotrophy may therefore have been a crucial event in the early history of life that made the biosphere less dependent on abiotic planetary processes (10–15). On a cellular level, the enzyme nitrogenase catalyzes the progressive reduction of molecular N_2_ to ammonia (NH_4_^+^). The most common, and generally considered most efficient nitrogenase isoform (16–18), the molybdenum/iron (MoFe)-bearing nitrogenase, uses eight electrons to convert one molecule of N_2_ into two molecules of NH_3_ and one molecule of H_2_ (1, 15, 19):


$$N_{2}\;+\;8\;FdH_{red}\;+\;16\;ATP\;+\;16\;H_{2}O\;\to \;2\;NH_{3}\;+\;H_{2}\;+\;8\;FdH_{ox}\;+\;16\;ADP\;+\;16\;P_{i}$$

As indicated in the equation above, adenosine tri-phosphate (ATP) is involved in the process to overcome the large activation energy of BNF, being converted to adenosine di-phosphate (ADP) and orthophosphate (P_i_). ATP is then regenerated elsewhere in the cell, which, in the case of oxygenic phototrophs, occurs largely during daytime photosynthesis. Three isoforms of nitrogenase have been identified to date, each containing an O_2_-sensitive catalytic domain Fe- sulfur (S) cluster and an active site FeScluster containing a heterometal cofactor of either FeMo, iron/vanadium (FeV), or FeFe (20). Despite their sensitivity to O_2_, nitrogenases, including the alternative nitrogenases, are encountered in prokaryotes with a range of respiratory requirements including aerobes, facultative anaerobes, and anaerobes (21, 22). All nitrogenases generate H_2_ during BNF, with the alternative isoforms of the FeFe- and VFe-bearing nitrogenases generating more H_2_ relative to NH_4_^+^, making them less efficient for N_2_ fixation, while placing additional energy requirements on the cell (14–16, 18, 19). All diazotrophs encode a MoFe-nitrogenase, with some encoding one or both alternative nitrogenases as well (20, 21). Alternative nitrogenases are only expressed under N- and Mo-depleted conditions, suggesting that they arose due to niche-driven metal availabilities (21).

Earlier phylogenetic reconstructions suggested that the nitrogenase enzyme, which catalyzes N_2_ reduction to NH_4_^+^, emerged relatively late, after the Great Oxidation Event (GOE) in the Paleoproterozoic (23, 24). This finding was reconciled at the time with the observation that the abundance of Mo in marine shales also increased after the GOE due to enhanced oxidative weathering on land and the subsequent growth of the marine Mo reservoir (25). Hence, the most common variety of nitrogenase, which requires Mo (24, 26), may have benefited from this geochemical transition. However, more recent phylogenetic studies with large data sets have pushed the origin of the MoFe-nitrogenase back to the Archean eon (11, 13, 22, 27) and possibly back to the last universal common ancestor (11, 28, 29), although still under debate (12, 30). The LUCA origin may be consistent with the idea that N_2_ reduction to NH_4_^+^ could have evolved from an abiotic process occurring on sulfide minerals to an enzymatic process driven by FeS clusters (31). Pi et al. (22), however, suggest that modern-day nitrogenases evolved from bacteria and spread into the Archaeal lineage, given that diazotrophic bacteria occur at higher numbers, in more diverse niches, than diazotrophic Archaea. Even though Archean Mo levels were minimal, likely in the lower nM range (32) compared to the 108 nM average in the modern ocean (33), they appear to have been high enough to enable the emergence of the nitrogenase enzyme (34). The notion that nitrogenases evolved in bacteria is supported by the fact that diazotrophic Archaea encode the bacterial Mo uptake transporters (ModABC), rather than the archaeal Mo transporter (WrpABC) (22). Moreover, of relevance to this study, it was recently hypothesized that the mosaic distribution of nitrogenase genes in the Cyanobacterial lineage reflects the loss of the relevant MoFe-nitrogenase genes originally encoded in the last common ancestor (35).

To further elucidate the question of when BNF emerged, several studies have looked at nitrogen isotope ratios (δ^15^N) in ancient sedimentary rocks that preserve organic matter. The two stable isotopes of nitrogen (^15^N ≙ 0.3663% of atmospheric N atoms, ^14^N ≙ 99.6337% of atmospheric N atoms) are fractionated during many biological metabolisms that take up a form of nitrogen from the environment. Chemical reactions invariably favor the lighter over the heavier isotope (36), such that a product will be depleted in ^15^N relative to a substrate.

Culturing experiments with modern cyanobacteria—the major N_2_-fixing organisms in the modern ocean—revealed fractionations (*ε* = δ^15^N_biomass_ – δ^15^N_N2_, where δ^15^N = [(^15^N/^14^N)_sample_/(^15^N/^14^N)_air_ – 1] × 1,000) of 0‰ to −2‰ relative to atmospheric N_2_ (37–40). Slightly larger fractionations down to −8‰ were observed in deletion mutants encoding only one of the so-called alternative nitrogenases, where either Fe or V replaced Mo in the reaction site (39, 41). In contrast, redox reactions involving NH_4_^+^ oxidation to nitrate (nitrification), nitrate reduction to N_2_ (denitrification), or dissimilatory nitrate reduction to NH_4_^+^ (DNRA) as well as partial assimilation of dissolved NH_4_^+^ into cells are associated with much larger fractionations, including pathways that generate mostly positive δ^15^N values (42). This framework underpins the use of isotope data from organic-rich rocks as a proxy for the type of nitrogen metabolism that occurred during the time of sediment deposition.

**Fig 1:**
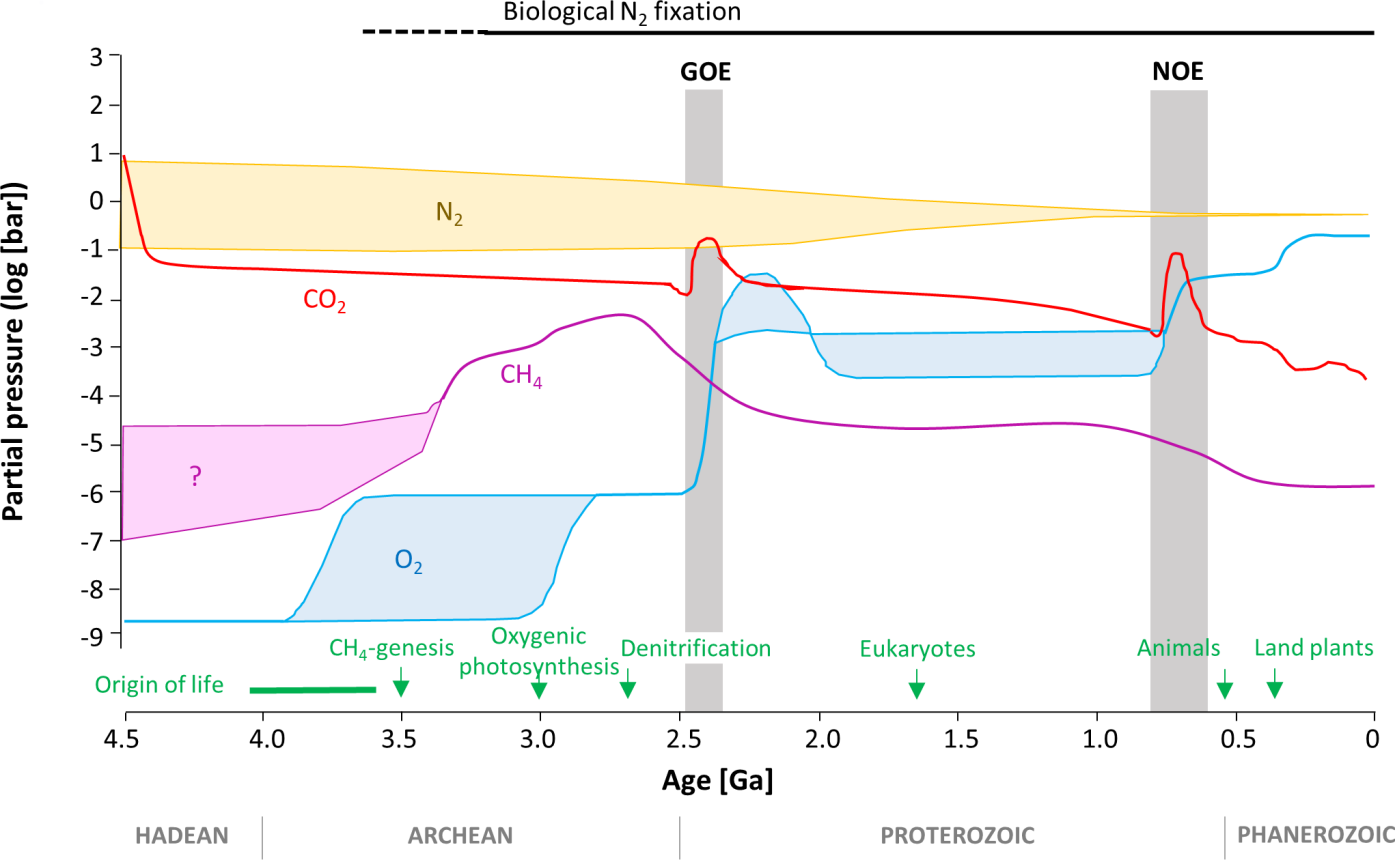
Atmospheric evolution through Earth’s history, showing the first geochemical evidence of key evolutionary processes of life. Redrawn and modified based on data from Goldblatt (51). Information about evolutionary events is reviewed by Lyons et al. (10). In the Archean (4–2.5 Ga), when biological N_2_ fixation evolved, *p*CO_2_ was probably two orders of magnitude higher than it is today, while *p*O_2_ was at least six orders of magnitude lower. GOE, Great Oxygenation Event; NOE, Neoproterozoic Oxygenation Event. The oldest geochemical evidence of free O_2_, thought to have arisen from oxygenic photosynthesis, is indicated.

In the mid-Archean, until about 2.8 billion years ago (Ga), many data points fall within the range of −2‰ to +2‰ (42–45), which is consistent with BNF driven by a MoFe-containing nitrogenase (42, 46). The geochemical data thus appear to confirm the conjecture that BNF originated early in Earth’s history. The necessary Mo may have been sourced from hydrothermal fluids (47, 48), from anoxic dissolution of volcanic glasses (49), or from low levels of oxidative weathering (50). However, a weakness in this line of argument has always been that the geochemical data are assessed against a small set of culturing studies, summarized in Denk et al. (38), which focused on a few select organisms. Furthermore, these experiments were conducted under standard modern atmospheric conditions, although the Archean atmosphere was enriched in CO_2_ and depleted in O_2_ relative to today (51) (Fig. 1). To our knowledge, the effect of these variables on nitrogen isotopic fractionation during diazotrophy has so far not been tested. High CO_2_ levels may spur biological productivity (52–55) and thus, perhaps increase the N-requirements of the cells. To meet the higher N-demand, diazotrophs may need to fix more N_2_, or recycle fixed N more efficiently within the cell, while lower O_2_ levels would make the FeS cluster in the nitrogenase less vulnerable to oxidation, therefore requiring less replacement synthesis. It is thus conceivable that the nitrogen isotopic fractionation responds to *p*CO_2_ and/or *p*O_2_, impacting the utility of δ^15^N as a biogeochemical proxy.

Here, we addressed some of these knowledge gaps with nitrogen isotope measurements of cyanobacterial Nostocales cultures grown under oxygenic photo-autrotrophic conditions in the laboratory under a set of atmospheric conditions, which mimic the pre-Cambrian Earth, present-day and future scenarios of *p*CO_2_ and *p*O_2_, under established experimental conditions (55, 56). Given that cyanobacteria evolved under conditions of elevated CO_2_ when compared to present-day levels, and without access to large amounts of biologically available N, we hypothesize that these organisms may respond positively to elevated CO_2_ levels and increase their N_2_ fixation rate with possible impacts of the isotopic inventory. Molecular clock analyses of the genomes of extant cyanobacterial species suggest that the heterocyst carrying genera of Cyanobacteria, the Nostocales, evolved around the time of the GOE, driven by increasing levels of atmospheric O_2_ (57, 58), further diversifying during the Proterozoic, when O_2_ levels were 1% of those of today (10). Furthermore, levels of O_2_ accumulating in non-diazotrophic cyanobacterial mats under an anoxic atmosphere reached levels comparable to those of the modern-day atmosphere (59), indicating that even under Archean conditions, diazotrophs may have been exposed to increased O_2_ levels in niches occupied by oxygenic phototrophs.

Cyanobacteria can tightly regulate their C/N balance [i.e., the ratio of total organic carbon (TOC) to total nitrogen (TN) in the cell] (60, 61), and therefore biomass C/N ratios were monitored to test if increased CO_2_ fixation alters the stoichiometry.

We further supplemented the culture data from this study with an extensive compilation of nitrogen isotopic fractionation data from an expanded data set reported in the literature including representatives of other species of Bacteria and Archaea, including examples of methanotrophy.

## MATERIALS AND METHODS

### Laboratory investigation

#### Strains and culture conditions

The cyanobacterial diazotrophs investigated in this study are listed in Table 1.

**TABLE 1:** Summary of the samples generated for isotopic analysis in this study^*a*^

Diazotrophs		Culture		Atmosphere		
Species	Strain	Medium	Format	PAL	eCO_2_	AnoxHC
*Nostoc* sp.	73.1 (DSM114167)	BG11_0_	Liquid	*n* = 9	*n* = 17	*n* = 4
		BG11_0_	Mat (basalt)	*n* = 3	*n* = 2	n.d.
			Mat (sand)	*n* = 3	*n* = 3	n.d.
*Calothrix* sp.	PCC7507	BG11_0_	Liquid	*n* = 3	*n* = 3	*n* = 5
*Nostoc* sp.	PCC7524	BG11_0_	Liquid	*n* = 3	*n* = 3	*n* = 5
*Nodularia spumigena*	CCY9414	BA_0_	Liquid	*n* = 3	*n* = 3	*n* = 3
*Nodularia spumigena*	NSBL06	BA_0_	Liquid	n.d.	n.d.	*n* = 4
*Nodularia harveyana*	SAG 44.85	BA_0_	Liquid	*n* = 3	*n* = 3	n.d.

^*a*^ In total, 82 samples from cultures of the diazotrophs listed below were investigated to assess the potential effect of changes in *p*O_2_ and *p*CO_2_, and culture format on δ^15^N fractionation. n.d., not determined.

*Calothrix* PCC7507 and *Nostoc* sp. PCC7524 were purchased from the Pasteur Culture Collection. *Nodularia spumigena* CCY 9414 was sourced from Lukas Stal (Culture Collection Yerseke, The Netherlands), *N. spumigena* NSBL06 was a gift from Hanna Mazur-Marsek (University of Gdańsk, Poland) and *Nodularia harveyana* SAG 44.85 was obtained from the Culture Collection of Algae at Göttingen University (SAG). *Nostoc* sp. 73.1 is a terrestrial cyanobacterium isolated from a coralloid root of a *Macrozamia* cycad (62, 63) and has been deposited in the German Collection of Microorganisms (DSMZ) as strain (DSM114167). Brackish dwelling *Nodularia* species were cultivated in Baltic Sea medium [Ba_0_, (see Table S4 at https://doi.org/10.5285/103983f8-b777-418c-9c4d-ba57065c91d5)] lacking combined nitrogen sources (55). Terrestrial *Nostoc* and *Calothrix* species were also grown under diazotrophic conditions in standard BG11_0_ (63) (Table S4). Media were replete with respect to Fe and Mo, containing 1.6 µM Mo, with BG11_0_ and Ba_0_ containing ~30 µM or ~24 µM Fe, respectively. Triplicate T_175_ ventilated cell culture flasks (Sarstedt, Germany) containing 100 mL of the appropriate medium were inoculated with 50 mL of a fresh cyanobacterial inoculum in the exponential phase. Flasks were laid flat to maximize gas exchange with the respective atmospheres and minimize shading effects. Experimental culture conditions were maintained as previously described (55, 56) under a high CO_2_ (eCO_2_) atmosphere comprising normal atmospheric O_2_ containing (20.95% O_2_) air supplemented to 2,000 ppm CO_2_ (Plant growth chamber E-22L, Percival, USA) to simulate climate change scenario, or an anoxic, Archean atmosphere comprising N_2_ gas supplemented with CO_2_ to 2,000 ppm with anoxic conditions maintained between 2 and 20 ppm O_2_, denoted as AnoxHC (GS MEGA 4 Glovebox, Germany). Control cyanobacteria were grown at present-day levels of CO_2_ (~400 ppm) and O_2_ (20.95%) defined here as the low CO_2_ (PAL) atmosphere (Plant growth chamber E-22L, Percival, USA). All cultures were acclimated to the alternative atmospheres for at least 4 months prior to setting up cultures for stable isotope analysis. Individual strains were cultured under identical conditions at 24°C, 60% humidity and 60 µmol photons m^−2^ s^−1^ with a 10:14 h light:dark cycle. Biomass was harvested after 3–4 weeks, when the cultures had reached stationary phase to maximize biomass accumulation and allow for comparative analysis with biomass obtained from the mat experiments, by centrifugation in sterile 50 mL Falcon tubes, washed two times with sterile MQ water and frozen at −80°C. After lyophilization for 48 h, aliquots of the dried biomass were sent for isotopic analysis.

Mats of *Nostoc* sp. 73.1 (DSM114167) were generated to test whether terrestrial cyanobacteria, with direct contact to the atmosphere, presented a different isotopic signature, compared to biomass generated in liquid medium. Beach sand (0.6–1 mm) was washed thoroughly with deionized water and sterilized overnight at 230°C. Basalt obtained from Eppelsberg Quarry was similarly treated. An 80 mL volume of sand, or basalt grains, was placed in a sterile deep Petri dish with ventilation knobs on the lid (Sarstedt, Germany). About 20 mL of BG11_0_ medium was pipetted onto the sand/basalt to moisten it. Thereafter, 5 mL of exponentially growing culture material was gently pipetted onto the sand/basalt and the closed Petri dishes were cultured as for the liquid cultures. The mats were harvested after a month, the sand/basalt grains brushed off and the biomass dried at 60°C for 48 h before being sent for isotopic analysis.

#### Isotopic analysis

Dry biomass was analyzed for nitrogen and carbon isotopes by flash combustion with an elemental analyzer (EA) IsoLink coupled via a ConFlo IV to a MAT253 isotope ratio mass spectrometer (Thermo Fisher Scientific). The EA was operated in dual-reactor mode, where one reactor (packed with chromium oxide granules), was held at 1,020°C, followed by silvered cobaltous cobaltic oxide granules as a sulfur trap. The second reactor, held at 650°C, was packed with copper wire to trap excess O_2_ and to convert NO_x_ species to N_2_ gas, followed by a water trap packed with magnesium perchlorate at room temperature. Ca 2–3 mg of freeze-dried, homogenized biomass was weighed into tin capsules and loaded into the autosampler of the EA, interspaced with blanks and standards with calibration standard USGS-40 and USGS-41 used for calibration (USGS-62 used for quality control). Data are expressed in delta notation [δ (‰) = *R*_sample_/*R*_standard_ – 1]. For nitrogen, *R* = ^15^N/^14^N, and the standard is atmospheric N_2_ gas. The average reproducibility of USGS-62 was ±0.2‰ for δ^15^N. To calculate the isotopic fractionation (magnitude of BNF relative to the nitrogen source), epsilon, that was used during the culturing experiments (*ε* = δ^15^N_biomass_ – δ^15^N_N2_), we also measured the isotopic composition of the N_2_ gas tank, using the dual inlet system of the same mass spectrometer, and it was found to have a composition of −10.43 ± 0.04‰. This value was used in the calculation of *ε* values following the equation above.

### Expanded data set (including literature review)

To address our research question, namely whether atmospheric CO_2_ and O_2_ levels influenced nitrogen isotopic fractionation during BNF by heterocytous cyanobacteria in liquid or mat cultures, available literature was screened, initiated by the seed publication of Denk et al. (38) and references therein. Pre-selection was done to exclude all scientific work reporting on inorganic/organic nitrogen-based growth of cyanobacteria, as well as stable isotope tracer experiments using enriched ^15^N-N_2_. To ensure the inclusion of naturally occurring organisms that came into direct contact with the respective atmospheric conditions, studies reporting on nitrogen fractionation during BNF of symbionts and/or artificially created mutants, were also excluded.

To further extend the data set with organisms other than cyanobacteria, publications reporting δ^15^N of organisms capable of BNF belonging to Bacteria, other than cyanobacteria, and Archaea were included in the survey. Data extraction from incorporated publications was done manually and/or upon provision by the authors themselves, and included species and strain information, atmospheric CO_2_ and O_2_ conditions, δ^15^N values, as well as abiotic meta-data (temperature, light, and salinity), and information pertaining to production of the hepatotoxin nodularin (if available). In case of deviating units, conversion to a common representation was performed (e.g., AnoxHC ≙ 0% O_2_). Calculation of *ε* from δ^15^N values was done as described above. The reference list of incorporated literature and tabular data are available in Tables S2 and S3 at https://doi.org/10.5285/103983f8-b777-418c-9c4d-ba57065c91d5.

We did not perform any statistical analysis of the effect of different nitrogenases on BNF ^15^N fractionation, because information on the type of nitrogenase occurring in a given diazotroph is frequently not reported in the specific scientific literature. In addition, we eliminated from our compiled data set any data from organisms where MoFe was absent, because large fractionation values have been reported for artificially created laboratory mutants of *Azotobacteria vinelandii* and *Rhodopseudomonas palustris* where the Mo based nitrogenase was deleted or inactivated (39, 41). Our study thus focuses only on organisms possessing the most commonly occurring MoFe nitrogenase, which enables us to focus on the effects of environmental parameters (rather than nitrogenase choice) on the isotopic fractionation.

### Statistical analysis

Spearman correlations were used to identify relationships between ε and environmental factors over different groups of organisms capable of BFN (Archaea, Bacteria, nd Cyanobacteria). Categorical, non-numerical parameters (toxin production and organism group) were translated to numerical groups to enable further analysis [e.g., 1 = nodularin production, 0 = non-nodularin production (see Tables S1 and S2 at https://doi.org/10.5285/103983f8-b777-418c-9c4d-ba57065c91d5)]. Correlations were considered significant when *P* was ≤0.05. Differences in *ε* for toxic and non-toxic cyanobacterial strains in the laboratory, as well as extended data set were analyzed using nonparametric Wilcoxon-Mann-Whitney-Test (represented by test score *T*) or parametric Student’s *t* test (denoted by test score *t*). Differences between organism groups (Archaea, Bacteria, and Cyanobacteria) and atmospheric conditions (PAL, AnoxHC, and eCO_2_) were analyzed using one-way ANOVA or Kruskal-Wallis one-way ANOVA on ranks followed by post hoc analysis (Dunn’s Method, Bonferroni *t* test). All statistical analyses were performed in Sigma Plot 13.0 (SYSTAT, Software Inc).

## RESULTS

### Laboratory investigation

**Fig 2:**
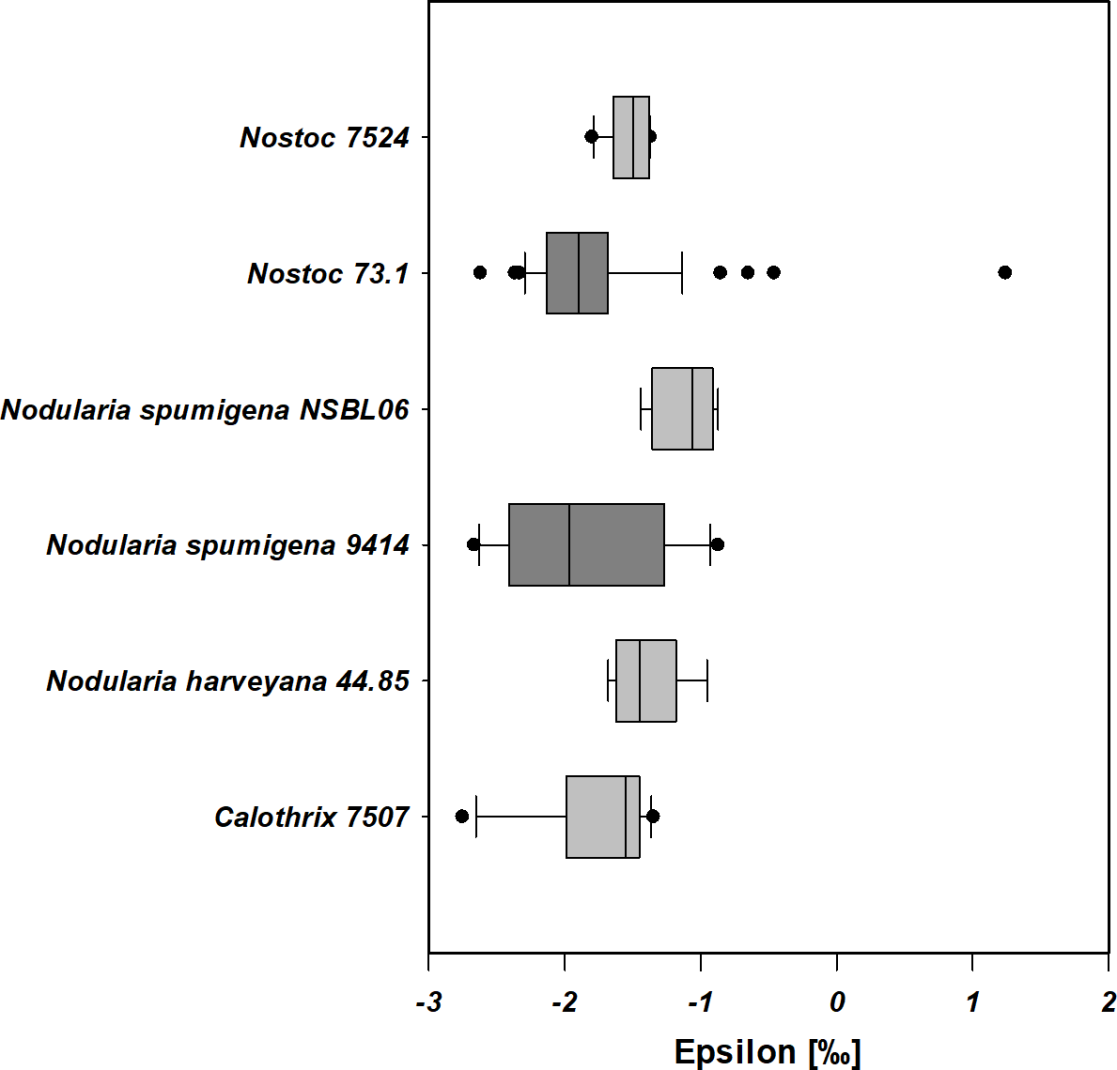
Box plots of epsilon observed in the laboratory during BNF for the different cyanobacteria investigated in this study. Dark gray box plots indicate toxin (nodularin) producing cyanobacteria, while light gray denotes non-nodularin-producing strains. The spread of the box encompasses the mean 50% of the data (= interquartile range) from the lower Q1 to the upper Q3 (25%–75%).

**Fig 3:**
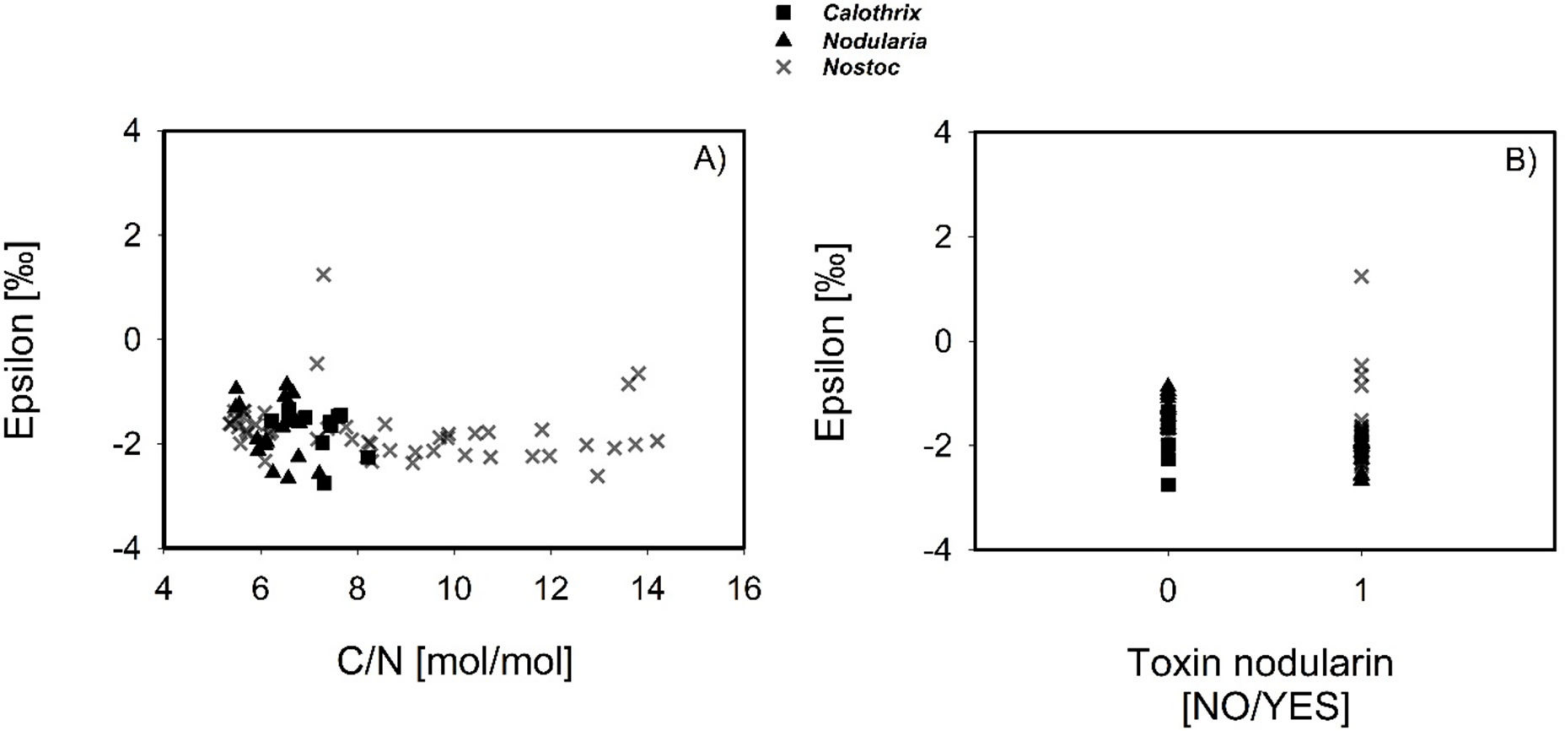
Correlation of epsilon with biomass C/N ratio and toxin profile for the species tested in the laboratory study. Values of ε displaying significant Spearman correlation coefficients from Table 2 are plotted against C/N ratio (**A**) and nodularin production score (**B**).

The isotopic fractionation factor *ε* measured in cyanobacterial species under laboratory conditions in this study ranged between 1.24‰ and −2.75‰ (Fig. 2; Table S1). There were significant correlations between *ε* values and C/N ratios (Spearman’s *ρ* = −0.326, *P* = 0.00439), as well as toxin production score (Spearman’s *ρ* = −0.514; *P* ≤ 0.001) (Table 2; Fig. 3), while no correlation was detected between *ε* and C/N ratios against *p*CO_2_ (eCO2 and AnoxHC ≙ 2,000 ppm, PAL ≙ 400 ppm) and/or *p*O_2_ (eCO2 and PAL ≙ 20.95%, AnoxHC ≙ 0%) across the whole data set. With increasing C/N ratio, *ε* decreases (Fig. 3A), as well as with increasing toxin scores (Fig. 3B). Correlation of *ε* and nodularin production was additionally verified by comparing mean values of both groups (toxic vs. non-toxic). The Mann-Whitney Rank-Sum Test indicates statistically significant differences (*T* = 1,834.000, *P* ≤ 0.001, *n* = 32/49) with lower *ε* values for toxin-producing strains (−1.88 ± 0.65‰) compared to non-toxic ones (−1.52 ± 0.35‰, Fig. 3B) across all *p*CO_2_ treatments.

**TABLE 2:** Spearman correlation of physical culture parameters versus epsilon determined in the laboratory investigation^*a*^

		Epsilon	C/N (mol/mol)	CO_2_ concentration	O_2_ concentration	Toxin (nod)
Epsilon	Correlation coefficient		**−0.355**	0.106	−0.065	**−0.564**
	*P* value		**0.001**	0.344	0.563	**<0.001**
	Number of samples		**81**	81	81	**81**
C/N (mol/mol)				0.003	0.201	**0.513**
				0.976	0.072	**<0.001**
				81	81	**81**
CO_2_ concentration						−0.090
						0.427
						81
O_2_ concentration						**0.329**
						**0.003**
						**81**

^*a*^ Information on the strains is provided in Table S1 (https://doi.org/10.5285/103983f8-b777-418c-9c4d-ba57065c91d5). Bold numbers indicate a significant correlation with minimum *P* ≤ 0.003.

Although no correlation of *ε* across the complete data set towards *p*CO*_2_* or *p*O*_2_* was found using Spearman’s correlation analysis, we detected differences in *ε* for the different *p*CO_2_ concentrations (400 ppm vs. 2,000 ppm) when considering toxin and non-toxin-producing cyanobacteria separately. First, looking at both groups of nodularin-producing cyanobacteria (toxic: *N. spumigena* CCY9414 and *Nostoc* sp. 73.1, and non-toxic: *N. spumigena* NSBL06, *N. harveyana* SAG44.85 and *Nostoc* sp. PCC7524) for which toxin status is known, significant differences in ε mean values for the two different *p*CO_2_ treatments can be found in non-toxic species (t = −3.597, *P* = 0.00192, *n* = 6/15), but not in toxin-producing strains (T = 502,000, *P* = 0.285, *n* = 18/31). Secondly, when analyzing *Nodularia* and *Nostoc* species independently, opposing trends were observed. *Nodularia* displayed a dependency of ε on *p*CO_2_ in the nodularin-producing strain, *N. spumigena* CCY9414 (T = 24,000, *P* = 0.024, *n* = 3/6), with lower values of ε at elevated *p*CO_2_ of 2,000 ppm at AnoxHC and eCO_2_, compared to 400 ppm at PAL (−2.37 ± 0.27 ‰ vs. −1.95 ± 0.03 ‰). No significant differences in ε mean values were detected for the toxic species *Nostoc* sp. 73.1 between the *p*CO_2_ treatments (T = 309,000, *P* = 0.978, *n* = 15/25), but rather in the non-toxic *Nostoc* sp. PCC7524 (t = −3.479, *P* = 0.007, *n* = 3/8).

### Expanded data set

**Fig 4:**
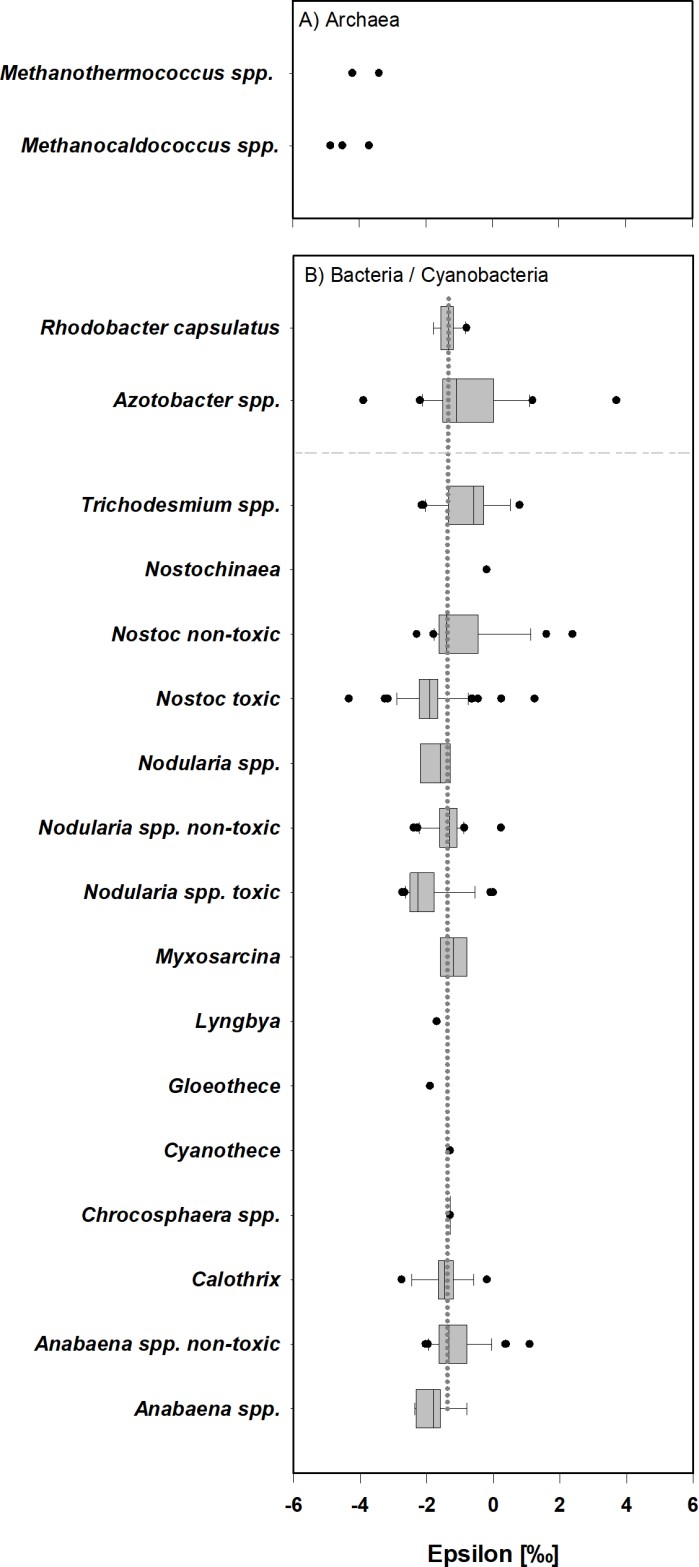
Box- and scatter plot (single data) displaying the isotopic fractionation factor (ε) for the combined set of diazotrophs. Data obtained from this study as well as literature data were categorized and plotted in three different organismal groups, namely (**A**) Archaea and (**B**) with Bacteria plotted above the dashed line, and cyanobacterial species below. Toxin-producing *Nostoc* and *Nodularia* spp. are plotted separately from known non-toxin producers. While some strains of *Anabaena* produce toxins, the toxin status of the species plotted as *Anabaena* spp. as well as *Nodularia* spp. without further specification, are unknown. *Anabaena variabilis* ATCC29413 (64) and *Anabaena cylindrica* PCC7122 (65) are non-toxin producers combined in *Anabaena spp*. non-toxic. For detailed information on the expanded data set see Table S2 at https://doi.org/10.5285/103983f8-b777-418c-9c4d-ba57065c91d5. The grey dotted line in panel B represents the mean value of ε (−1.38 ± 0.95 ‰) across all bacterial, including cyanobacteria, data points.

**Fig 5:**
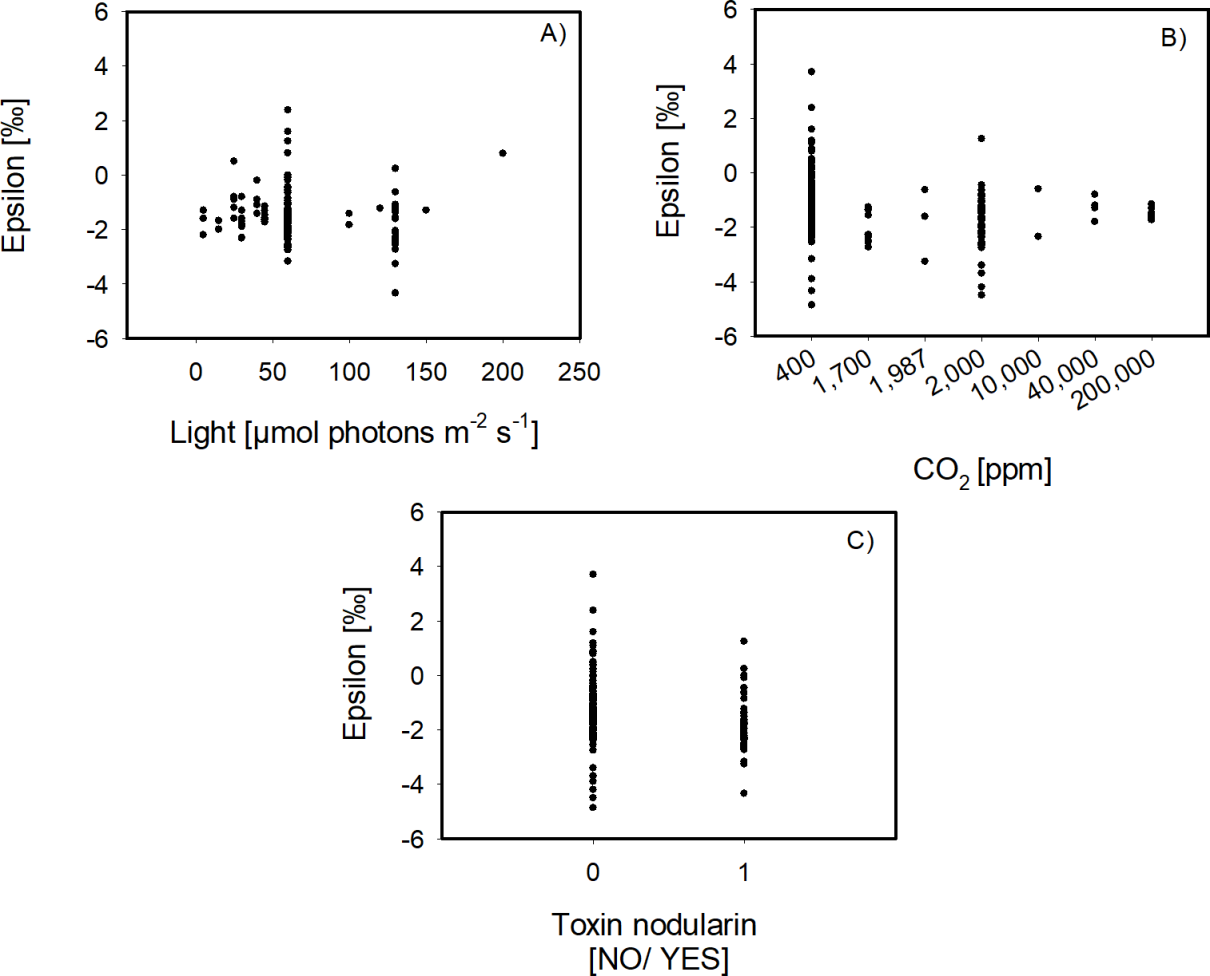
Representation of the correlation of epsilon to environmental variables in the expanded data- set. Epsilon values with significant Spearman correlation coefficients from Table 3 were plotted to highlight the correlation to light (**A**) and CO_2_ (**B**) and the toxin nodularin score (**C**) for the expanded data set.

Isotopic fractionation for the two different organism-groups revealed significant differences in ε for Archaea versus Bacteria/ Cyanobacteria, with mean values of −4.13 ± 0.53 ‰ and −1.38 ± 0.95‰, respectively (*P* < 0.001, *n* = 5/256, Fig. 4). Significant correlations of ε were detected with light (Spearman’s ρ = −0.176, *P* = 0.0197, *n* = 176) and *p*CO_2_ (Spearman’s ρ = −0.209, *P* = 0.000715, *n* = 261), as well as toxin production score (Spearman’s ρ = −0.436, *P* = 6.639E-014, *n* = 261; Table 3). With increasing light, *p*CO_2_ and toxin score, the ε values decrease (Fig. 5A through C). To further analyze the dependency of ε on *p*CO_2_, also in combination with toxin production, we statistically compared mean values of sub-groups of the expanded data set.

**TABLE 3:** Spearman correlation of parameters of the combined data sets, including literature values^*a*^

		Epsilon	Temperature	Salinity	Light	CO_2_ concentration	O_2_ concentration	Toxin (nod)
Epsilon	Correlation coefficient		0.122	−0.015	**−0.176**	**−0.203**	0.117	**−0.428**
	*P* value		0.055	0.826	**0.020**	**0.001**	0.059	**3.555E-013**
	Number of samples		247	215	**176**	**261**	261	**261**
Temperature				**−0.296**	−0.030	0.090	**−0.249**	**−0.281**
				**<0.001**	0.687	0.156	**<0.001**	**<0.001**
				**214**	179	250	**250**	**250**
Salinity					**0.290**	**−01.49**	0.103	−0.055
					**<0.001**	**0.028**	0.128	0.432
					**175**	**218**	218	218
Light						0.045	0.136	**0.307**
						0.554	0.070	**<0.001**
						179	179	**179**
CO_2_ concentration							**−0.685**	**0.156**
							**<0.001**	**0.011**
							**264**	**264**
O_2_ concentration							s	**0.141**
								**0.022**
								**264**

^*a*^ Information on species and extracted data is provided in Table S2 (https://doi.org/10.5285/103983f8-b777-418c-9c4d-ba57065c91d5). Bold numbers indicate a significant correlation with minimum *P* ≤ 0.05. C/N is not included as these values were largely not reported.

**Fig 6:**
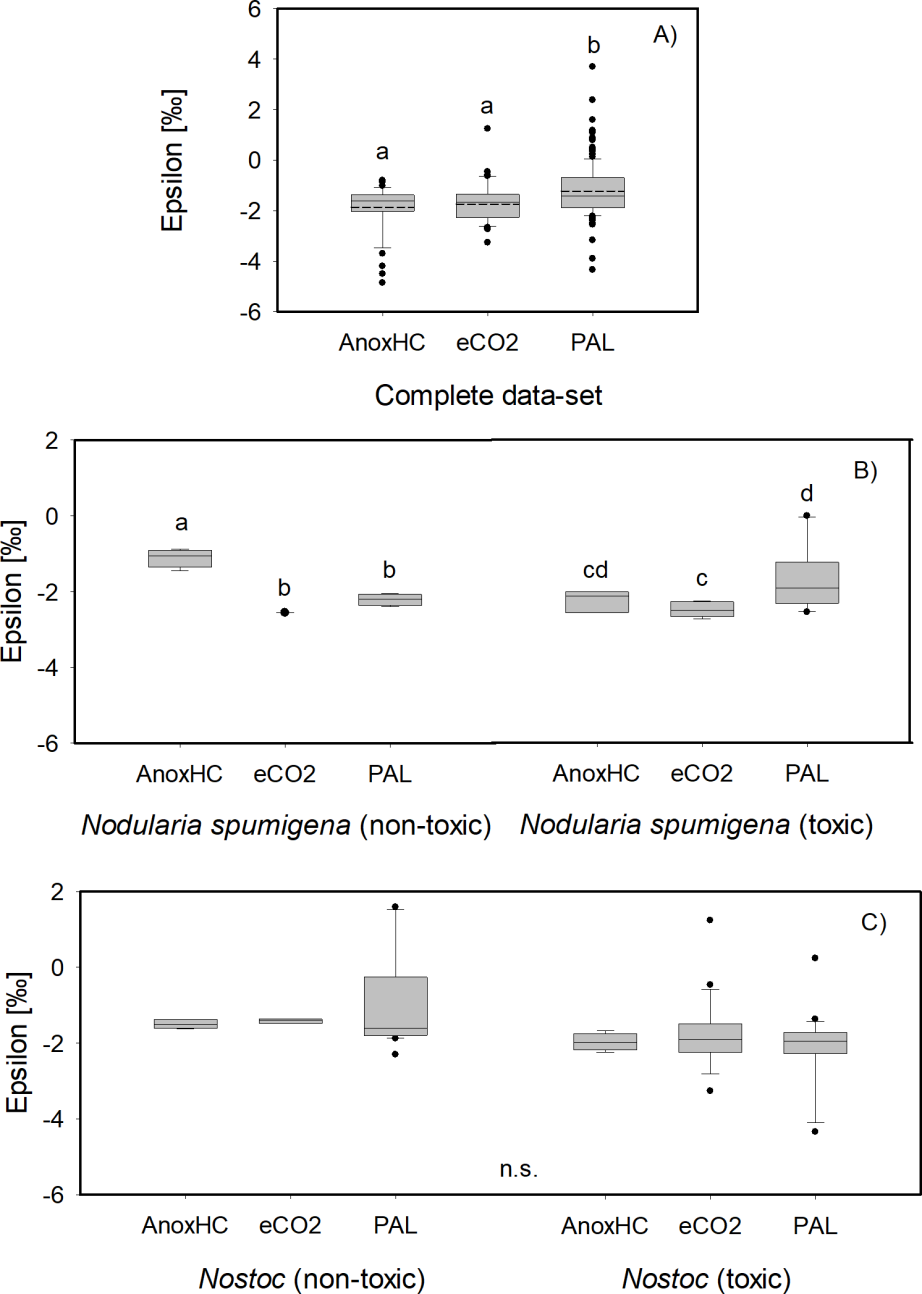
Box plot of epsilon for the different *p*CO_2_ atmospheres of the expanded data set (**A**) as well as for *Nodularia spumigena* (**B**) and *Nostoc* spp. (**C**) specifically. Dashed-dotted line in (A) represents the mean value. Different letters in the separate figures denote significant differences (*P* > 0.05) in *ε* among the three tested atmospheric conditions (AnoxHC, eCO2, and PAL) as determined by Duncan’s post hoc ANOVA statistical analysis for the whole expanded data-set (**A**) and non-toxic and toxic *N. spumigena* strains (**B**). Note that no significant differences were detected in non-toxic and toxic *Nostoc* strains (n.s., **C**). For detailed information on the expanded data set see Tables S2 and S3 at https://doi.org/10.5285/103983f8-b777-418c-9c4d-ba57065c91d5.

Firstly, significantly lower ε values, within 1 ‰, were detected for the whole data set under eCO_2_ (177–2,000 ppm, −1.74 ± 0.79 ‰) and AnoxHC (2,000 ppm to 20%, −1.87 ± 0.91 ‰) compared to present-day PAL (400 ppm, −1.21 ± 1.05 ‰) (eCO_2_ versus PAL *P* = 0.002, *n* = 48/166, AnoxHC versus PAL *P* = 0.013, *n* = 47/166, Fig. 6A, see also Table S3 at https://doi.org/10.5285/103983f8-b777-418c-9c4d-ba57065c91d5), but overall differences in ε fall within the analytical uncertainty of 0.2 ‰. Secondly, *p*CO_2_ effects on ε of nodularin-producing cyanobacteria were analyzed independently. For *Nodularia*, non-toxic strains exhibited significantly lower epsilon mean values under eCO_2_ (−2.55‰, single value) and PAL (−2.21 ± 0.15‰) conditions, when compared to AnoxHC (−1.11 ± 0.24‰) conditions, with *t* = 7.750, *P* < 0.001, *n* = 4 for PAL versus AnoxHC (Fig. 6B; Table S3). For the toxic strain of *N. spumigena* CCY9414*,* significantly lower mean values of *ε* were observed under eCO_2_ (−2.48 ± 0.17‰) compared to PAL conditions (−1.63 ± 0.79‰; Fig. 6B). The mean value of *ε* for AnoxHC (−2.23 ± 0.23‰) was not significantly different from the two other *p*CO_2_ conditions (Table S3). No significant differences in *ε* were recorded for *Nostoc* species under the three *p*CO_2_ conditions, regardless of toxin production score (Table S3). Mean *ε* values for non-nodularin-producing strains were −1.49 ± 0.11‰ for AnoxHC, −1.42 ± 0.05‰ for eCO_2_ conditions and −0.89 ± 1.23‰ for PAL (Fig. 6C). For toxin-producing strains, mean values of *ε* were −1.97 ± 0.20‰ for AnoxHC and −1.75 ± 0.91‰ for eCO_2_, as well as −2.06 ± 0.88‰ for PAL (Fig. 6C).

## DISCUSSION

Overall, the range of C/N and *ε* remains relatively narrow. In our laboratory cultures, *ε* values span a range of 3.99‰ ranging from 1.24‰ to −2.75‰. In the extended data set, including 23 published records from the Archaea and Bacteria, along with cyanobacteria, *ε* spans a wide range of 8.56‰, ranging from 3.7‰ to −5.86‰ with a mean of −1.44 ± 0.89‰ for all cyanobacteria. Although we found statistically significant differences in *ε* in-between the atmospheric treatments, the overall differences were relatively small keeping the 0.2‰ analytical uncertainty of determination in mind.

Some of the most negative values were recorded in MoFe-nitrogenase encoded by hyperthermophilic N_2_-fixing methanogens (Fig. 4), namely *Methanocaldococcus jannaschii* at 90°C (66), *Methanocaldococcus* at 85°C and *Methanothermococcus* at 55°C (67). This suggests a potential albeit non-significant (*P* = 0.055) correlation of temperature on *ε* values and motivates future investigations expanding the effect of temperature on biomass δ^15^N, beyond the 24°C used in our study.

### What causes ^15^N/^14^N fractionation in N_2_ fixation on a cellular level?

Several studies have investigated the chemical reaction pathway of biological N_2_ reduction to NH_4_^+^ and its effects on δ^15^N, which provide an important context for our data indicating a 4‰ variability. When Sra et al. (68), investigated the isotopic discrimination of the Mo-nitrogenase *in vitro*, that is, after isolation from a living organism, they measured an isotopic fractionation (*ε* = δ^15^N_Mo-nitrogenase_ − δ^15^N_N2_) of −17‰, which was interpreted as a kinetic effect. This fractionation is much larger than the values inferred from N_2_-fixing microorganisms (38) (see Table S2 at https://doi.org/10.5285/103983f8-b777-418c-9c4d-ba57065c91d5), which led Unkovich (69) to propose that the reduction step from N_2_ to NH_4_^+^ is not the rate-limiting step in the process. Instead, the diffusion of N_2_ into the cell and/or the utilization of the NH_4_^+^ product downstream of the nitrogenase enzyme are likely rate-limiting, such that the large kinetic effect measured by Sra et al. (68) in an *in vitro* system is not expressed *in vivo*. This may be different in the VFe- and FeFe-based nitrogenases, the so-called alternative nitrogenases, where the reduction step is less efficient, such that it can become rate-limiting (69). This model was supported through culturing experiments with varying N_2_ partial pressures (*p*N_2_), which showed a decrease in the absolute value of *ε* by up to 0.6‰ as *p*N_2_ was changed from 0.8 to 0.1 bar (70). At low *p*N_2_, the supply of N_2_ by diffusion into the heterocyte appears to become limiting, such that the isotopic fractionation associated with the reduction step has even less net impact. Limited diffusion of N_2_ and O_2_ in heterocytous strains of cyanobacteria results from thickened cell walls of the specialized nitrogen-fixing cells, thereby reducing the O_2_ exposure of the nitrogenase simultaneously also restricting diffusion of other gases such as N_2_, CO_2_, and H_2_, a by-product of BNF (15, 70).

Increases in δ^15^N were observed under Fe limitation in the cyanobacterium, *Anabaena variabilis* (71), while neither Mo and phosphate levels, nor growth phase, nor heterotrophy influenced the signal (41, 71). If N demands are met through BNF, excess N is often released into the environment (39). These nitrogen compounds can show varying isotopic composition depending on the growth stage of organisms (72), as well as physiological processes involved (e.g., assimilation, deamination, and transamination) (73, 74). Using the non-cyanobacterial *Azotobacter vinelandii*, a decrease in δ^15^N fractionation of biomass of +1.2‰ for the MoFe-nitrogenase was observed. Furthermore, irrespective of which nitrogenase was active, secreted biomass, including the Fe scavenging molecules, siderophores, were lower in δ^15^N than the associated biomass (39).

Isotopic fractionation furthermore occurs when the end-product of BNF, NH_4_^+^, is incorporated into carbon skeletons by the glutamate dehydrogenase and subsequent transfer of the amino group by a transaminase, such as glutamine synthetase/glutamate synthase (GS/GOGAT) (75, 76). Finally, N-containing compounds such as proteins, nucleotides, porphyrines, and proteoglykanes, but also individual amino acids, may differ in respect to their δ^15^N values, providing an explanation for the <1‰ variability in δ^15^N we observe within toxin and non-toxin-producing cyanobacteria. The production rate of these end products ultimately depends on the growth rate of the cell and thus links the nitrogen to the carbon cycle, and ultimately to atmospheric *p*CO_2_ in the oxygenic photosynthesis in cyanobacteria. In summary, the observation that isotopic fractionations occur during N_2_-uptake into the cell and during further processing of fixed nitrogen into organic molecules motivates our search for dependencies between *ε* and environmental variables that impact biological N-demand.

### Is there any risk that the presence of VFe and/or FeFe nitrogenase is perturbing our data?

Examples from the literature of organisms producing either an additional VFe-based (*vnf* encoded) or FeFe-based (*anf* encoded) nitrogenase, or both, exist; however, no example has been identified to date that encodes either of these nitrogenases in the absence of the MoFe-containing nitrogenase (*nif* encoded) (12, 18, 77). Additionally, the MoFe-nitrogenase is preferentially expressed under Mo replete conditions, with the VFe-nitrogenase only expressed under Mo-depleted diazotrophic conditions in *A. variabilis* ATCC29413 (64) and *Azotobacter vinelandii* (78), the latter below 25 nM Mo, with V concentrations as low as 8 nM (79, 80). The lowest levels of Mo are encountered in rivers, freshwater lakes and estuaries (33), as well as in tropical soils (81), with the modern ocean Mo levels ranging from 2 to 108 nM (33, 71, 82). Given the 2–3 ppm Mo in the Earth’s crust (83), with some strains like *A. variabilis* able to extract Mo from silicates using metallophores (84), BNF organisms are thought to be able to access sufficient Mo to meet their needs (85). Mo at 10 and 1 µM also stimulated BNF in the archaeon, *Methanosarcina barkerei* 227, in the absence of NH_4_^+^ or glutamate (86). Collectively, these observations suggest that MoFe nitrogenase is used preferentially in biology unless organisms are under severe Mo stress (20, 21, 26), which was not the case in our laboratory experiments. Furthermore, ensuring that an experimental setup is Mo-free is challenging and even cultures with just a few nM Mo have been reported to perform N_2_ fixation (82). Therefore, even though some of the strains reported in the literature in this study do contain VFe and/or FeFe nitrogenase in addition to MoFe nitrogenase, we consider it unlikely that the trends in the data are impacted by the presence of these enzyme variants. This allows us to focus our discussion on the effect of environmental variables on *ε* and C/N ratios.

### Potential effects of *p*CO_2_ and *p*O_2_ on ε

Having established that the presence of VFe or FeFe nitrogenase does likely not impact the trends in our data, we can now turn to the investigation of environmental parameters on ε during BNF. While the effects of atmospheric N_2_ levels on *ε* were previously investigated (70), the potential influence of how changes in *p*O_2_ and *p*CO_2_, as well as other environmental parameters such as light, temperature, and salinity, has not.

First, our study identified a significant correlation between *ε* and C/N content of the cultured biomass (Table 2) with decreasing *ε* at increasing C/N ratios. In other words, isotopic fractionations become slightly larger as the bulk biomass becomes depleted in nitrogen. This observation may indicate that the incorporation of fixed N into biomass within the cell creates a minor bottleneck that imparts a small isotopic fractionation. Second, statistically significantly lower *ε* values were detected in the expanded data set at elevated *p*CO_2_ of ≥2,000 ppm, regardless of the *p*O_2_. This may be consistent with the idea that high *p*CO_2_ stimulates the growth rate in C-fixing cyanobacteria (55), and hence, N-demand, exacerbates the bottleneck of N incorporation into biomass. This conclusion would be consistent with the current understanding of N-isotopic fractionation during BNF described above.

Third, both our laboratory investigation and the expanded literature data identified species-specific responses to atmospheric *p*O_2_ and *p*CO_2_ levels (Tables 2 and 3). Specifically, *N. spumigena* CCY9414 (marine) exhibited significantly lower ε values when grown at 2,000 ppm CO_2_, irrespective of *p*O_2_, while *Nostoc* sp. PCC7524 (freshwater) showed lower isotopic fractionation (less negative *ε* values) under elevated CO_2_ conditions. This observation suggests that marine diazotrophs may react differently to increasing atmospheric CO_2_ levels than terrestrial or freshwater species. The difference in response between marine and freshwater organisms may be due to different environmental adaptations. Seawater is buffered by HCO_3_^−^ with a relatively constant pH leading to relatively constant CO_2_ supplies, whereas freshwater pH and dissolved CO_2_ may vary widely (87). It is conceivable that freshwater organisms have developed strategies to maintain stability across natural environmental CO_2_ fluctuations while marine organisms lack this skill, making them more sensitive to external perturbations.

In line with this observation, the aquatic *Nodularia* species, including *N. spumigena* CCY9414, demonstrated a sixfold higher biomass accumulation under high CO_2_ conditions when compared to terrestrial *Nostoc* species (55). This was accompanied by a 17-fold increase in BNF and raised PON content compared to the *Nostoc* spp., providing support for increased nitrogen demand to maintain cellular C/N ratios under high CO_2_ conditions, as recorded for *N. spumigena* sp. KAC12 (88) and *N. spumigena* CCY9414 (54). C/N ratios were only slightly increased under increased CO_2_ levels in *Cyanothece* sp. ATCC51142, *N. spumigena* sp. IOW-2000/1 and *Calothrix rhizosoleniae* SC01 (89). Species phenotypic plasticity must also be considered as *Nostoc punctiforme* CPCC41, grown at 940 ppm CO_2_ demonstrated an increase in BNF rate (90) and *Dolichospermum circinale* appeared to benefit from raised CO_2_ levels of 1,700 ppm (91) in terms of elevated biovolume and chlorophyll a content. The recorded positive growth effects of elevated *p*CO_2_ on cyanobacteria most likely arises from downregulation of the carbon concentrating mechanisms (92–95), which are needed in most phytoplankton species to compensate for the low efficiency of the photosynthetic enzyme ribulose-1,5-bisphosphate carboxylase oxygenase (RuBisCo) under present-day CO_2_ levels (96). The C/N ratios and *ε* values were, however, not determined in the abovementioned studies.

Finally, the lack of correlation between *ε* values and *p*O_2_ likely attests to the ability of diazotrophs to protect the nitrogenase enzyme from atmospheric O_2_ (15, 93–95, 97). In our simulated Archean environment, low external O_2_ should not have been a limiting growth factor, as O_2_ is produced from the hydrolysis of water during oxygenic photosynthesis during the day, and from respiration during the night (15, 56). In fact, O_2_ release rates were higher in the anoxically grown, non-diazotrophic, filamentous *Pseudanabaena* PCC7367 than in an O_2_-rich modern-day atmosphere (56). Increased O_2_ levels are argued to have placed stress on early Cyanobacteria through increased levels of O_2_-free radicals, and hence protein degradation. Thus, one could argue that under reduced, micro-oxic conditions, there would be less protein damage, and hence a lower N demand, requiring less N_2_-fixation. How this impacted the δ^15^N signal had not been investigated until this investigation.

### Proposed impact of nodularin production on internal nitrogen fractionation

Although not within the scope of our initial hypothesis of *p*O_2_ and *p*CO_2_ affecting nitrogen isotopic fractionation during BNF, an unexpected correlation of *ε* to nodularin production was observed. Our laboratory investigation (Fig. 2; Table 2), identified significantly larger fractionations (more negative *ε* values) in the nodularin-producing strains of *N. spumigena* CCY9414 and *Nostoc* sp. 73.1, compared to the non-nodularin-producing strains of the same species. This observation was maintained in the expanded data set with toxin-producing *Nodularia* and *Nostoc* strains having significantly lower mean *ε* values compared to known non-toxin-producing strains of cyanobacteria (Fig. 4; Table 3). Our literature analysis suggests that some *Anabaena* species, for which toxin production is not known, may indeed be toxin producers, given their low *ε* values compared to the overall mean, indicating higher δ^15^N fractionation. The *Anabaena* strains of *Anabaena variabilis* (64) and *Anabaena cylindrica* (65) are known not to produce toxins. They also did not exhibit increased fractionation of nitrogen (Fig. 4), supporting our hypothesis that toxin production is involved in internal N-cycling, resulting in lower *ε* values.

Siderophores are generally synthesized through non-ribosomal synthesis mechanisms, the same process used to synthesize the toxin nodularin. How this may impact the δ^15^N of excreted material from toxin-producing cyanobacteria remains to be determined. Cyanobacterial toxins overall (i.e., cyclic peptides, alkaloids, and polyketides/polycyclic derivates), are located intracellularly, being released upon cell death during bloom decay (98). Additionally, they are often associated with proteins during synthesis and stress situations (99). The causal connection between nodularin production and increased fractionation of ^15^N of <1‰ is speculative so far. At least a strong correlation of the production of the N-rich (C/N ratio lower than Redfield ratio 6.6) secondary compound nodularin is undisputed, and this secondary metabolite may be removing an isotopically fractionated (^15^N-enriched) nitrogen reservoir from the bulk biomass. Nodularin is a cyclic pentapeptide with five amino acids in the peptide ring. It is the product of a secondary metabolic pathway comprised of mixed polyketide synthases and non-ribosomal peptide synthetases (100). The purpose of nodularin production is not fully resolved, but because of the high-energy demand of nodularin production, an ecological or physiological advantage over non-toxin producers must be assumed. A function within the cell metabolism is likely, as a rapid and covalent binding of nodularin to proteins of unknown function upon abiotic stress, was shown (99). In addition, nodularin and microcystin (a related cyclic heptapeptide) might function as signaling or regulatory proteins (101). The strong connection of microcystin and nodularin turnover with the cellular N-cycle (102) is indicated by the regulation of their synthesis by the global N uptake regulator NtcA (103, 104), which activates the transcription of nitrogen assimilation genes (105, 106). The difference in cellular response to *p*CO_2_ and toxin production status is highlighted in Fig. 6B and C. Nodularin-producing *N. spumigena* shows a significantly larger fractionation under high CO_2_ levels, whereas the non-toxin-producing strains do not. This is interesting, as aquatic *Nodularia* species overall exhibited increased BNF rates under the same *p*CO_2_ conditions (55), suggesting that toxin production has some effect on internal nitrogen cycling.

While nodularin-producing *Nostoc* species also exhibited similar *ε* values compared to toxic *Nodularia* species, (Fig. 4; Table 3), isotopic fractionation was less affected by changing levels of *p*CO_2_. Previously, *Nostoc* spp. demonstrated significantly reduced BNF rates when compared to *Nodularia* species, under both low and high *p*CO_2_ conditions, suggesting species-specific constraints on BNF, possibly related to habitat differences (aquatic vs. terrestrial) (55).

It is currently unknown when biological toxin production evolved. However, it is conceivable that its isotopic effect of driving the biomass to slightly more negative δ^15^N values may have impacted the geological record. This aspect is worth further investigation.

There are chemical and physical processes, which contribute to toxin removal and degradation such as photolysis in the upper water layer (98), adsorption to particles and sedimentation (107) or enzymatic breakdown (108). Whether the toxin δ^15^N imprint is preserved within the bulk biomass of such a complete ecological system, having undergone mineralogical processing during preservation, is unknown.

### Implications

**Fig 7:**
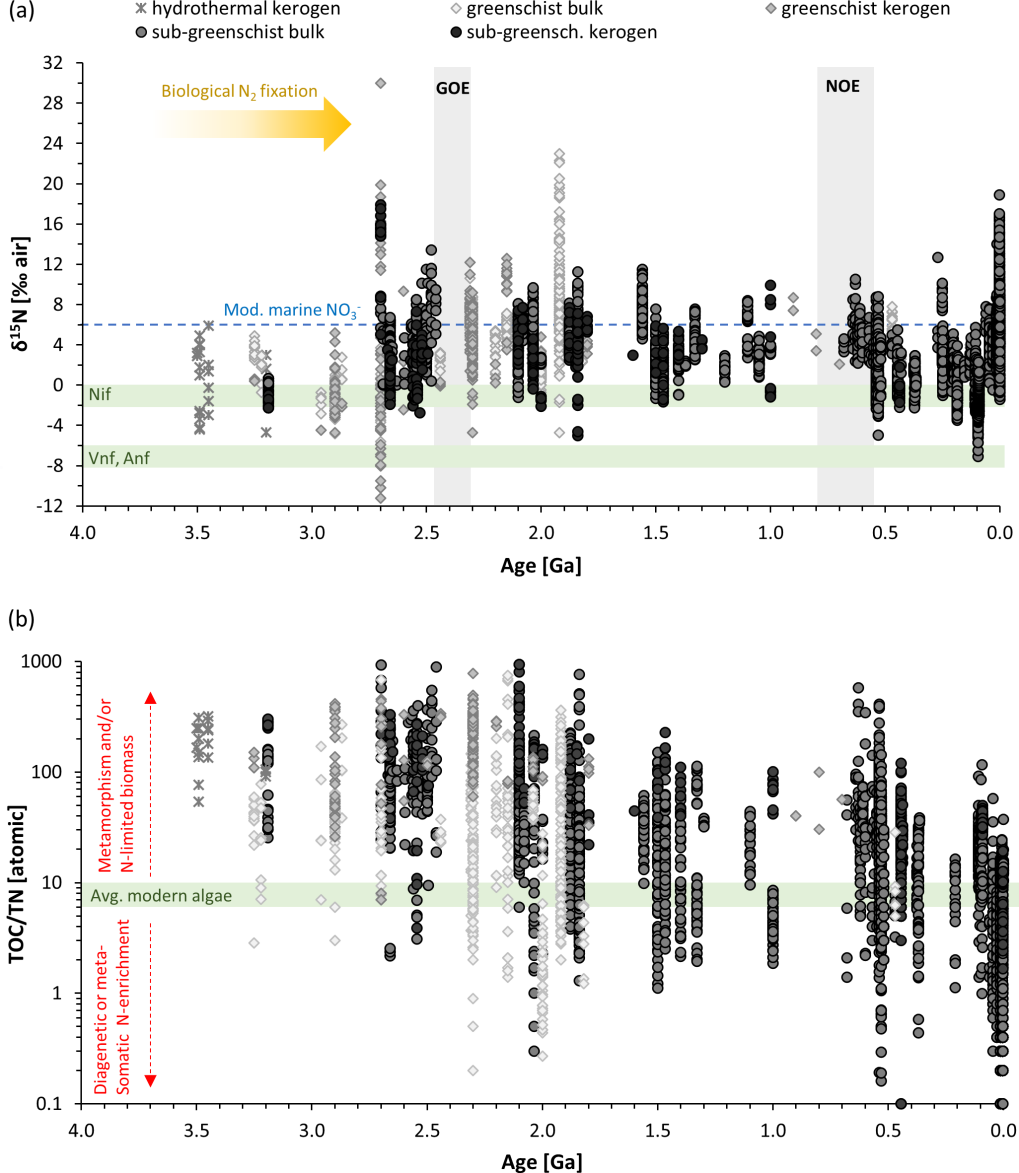
Geochemical data from the sedimentary rock record. (**a**) Nitrogen isotopes in bulk rocks and kerogen isolates (organic matter extracted from the bulk rock with hydrofluoric acid). (**b**) Ratios of organic carbon to total nitrogen. In both panels, samples are separated by metamorphic grade, and samples of amphibolite grade or higher are excluded, because such high metamorphic alteration is likely to have perturbed the primary signature by more than 2‰ [reviewed by reference (112)]. Plots are generated based on data from Johnson and Stüeken (113).

Generally, the range in ε detected in our laboratory and expanded data set is small compared to fractionations of well over 10‰ observed for biological metabolisms that involve large reservoirs of dissolved ammonium, nitrite, or nitrate (109). Although there are correlations between *ε*, C/N ratios, and *p*CO_2_ that likely relate to biological N processing within the cell, the variance in *ε* remains only within a few permil. Since the isotopic composition of atmospheric air has likely not changed much (<2‰) since the early Archean (110), the *ε* values reported here for BNF, where the substrate is atmospheric N_2_, are equal to the δ^15^N value of biomass that would be archived in the sedimentary rock record. Comparing our range of *ε* values (i.e., biomass δ^15^N for diazotrophs) to sedimentary rocks over the past several billion years shows that the effects of C/N and *p*CO_2_ would likely not be resolvable (Fig. 7). Diagenetic and metamorphic alteration may additionally bias sedimentary δ^15^N values by a few permil compared to the composition of the originally buried biomass, complicating the interpretation of the sedimentary δ^15^N values (111, 112). On the contrary, the relatively low sensitivity of *ε* to *p*CO_2_ and C/N ratios implies that the δ^15^N signal remains a reliable indicator of BNF in deep time, at least given the current knowledge base. However, it is important to note that current data of *ε* during BNF remain strongly biased towards cyanobacteria. More δ^15^N information should be obtained for other nitrogenase-containing organisms including Archaea and the Bacteroides, Chlorobi, Firmicutes, and Verrucomicrobia, with more representation from the Proteobacteria (26, 77). As shown by the extended data set, (hyper-)thermophilic methanogens in particular, appear to express larger *ε* values for BNF, suggesting that higher temperatures encountered, for example, in hot springs, may induce greater fractionation, possibly resulting from increased internal N-cycling.

Importantly, we emphasize the unexpected finding that toxin-producing organisms express slightly larger fractionations (more negative *ε* values) compared to non-toxin producers. The antiquity of toxin production is unknown, but our finding opens the intriguing possibility that the δ^15^N record may, perhaps in conjunction with phylogenetic reconstructions, provide information about the onset of this metabolic trait.

## Data Availability

All data generated in this project are available from the National Geoscience Data Centre of the British Geological Survey (DOI: 10.5285/103983f8-b777-418c-9c4d-ba57065c91d5) under https://doi.org/10.5285/103983f8-b777-418c-9c4d-ba57065c91d5.

## EDITOR'S NOTE

This article was handled by Betül Kaçar, who acted as a Guest Editor in consultation with the Editor in Chief, Gemma Reguera.
